# Design of a colicin E7 based chimeric zinc-finger nuclease

**DOI:** 10.1007/s10822-014-9765-8

**Published:** 2014-06-22

**Authors:** Eszter Németh, Gabriella K. Schilli, Gábor Nagy, Christoph Hasenhindl, Béla Gyurcsik, Chris Oostenbrink

**Affiliations:** 1 Department of Inorganic and Analytical Chemistry, University of Szeged, Dóm tér 7, Szeged, 6720 Hungary; 2 Institute of Molecular Modeling and Simulation, University of Natural Resources and Life Sciences (BOKU), Muthgasse 18, 1190 Vienna, Austria; 3 Christian Doppler Laboratory for Antibody Engineering, Department of Chemistry, Vienna Institute of BioTechnology, University of Natural Resources and Life Sciences (BOKU), Muthgasse 18, 1190 Vienna, Austria; 4 MTA-SzTE Bioinorganic Chemistry Research Group of Hungarian Academy of Sciences, Dóm tér 7, Szeged, 6720 Hungary

**Keywords:** Metalloenzyme, Zinc finger nuclease, Colicin E7, Computational protein design

## Abstract

Colicin E7 is a natural bacterial toxin. Its nuclease domain (NColE7) enters the target cell and kills it by digesting the nucleic acids. The HNH-motif as the catalytic centre of NColE7 at the C-terminus requires the positively charged N-terminal loop for the nuclease activity—offering opportunities for allosteric control in a NColE7-based artificial nuclease. Accordingly, four novel zinc finger nucleases were designed by computational methods exploiting the special structural features of NColE7. The constructed models were subjected to MD simulations. The comparison of structural stability and functional aspects showed that these models may function as safely controlled artificial nucleases. This study was complemented by random mutagenesis experiments identifying potentially important residues for NColE7 function outside the catalytic region.

**Electronic supplementary material:**

The online version of this article (doi:10.1007/s10822-014-9765-8) contains supplementary material, which is available to authorized users.

## Introduction

Zinc finger nucleases (ZFN) hydrolyze DNA at a specific sequence. Three or four zinc finger (ZF) units are responsible for the site-specific DNA-binding, and in most ZFN-s a FokI nuclease domain linked to the ZF-s cleaves DNA [1, 2]. One ZF-domain recognizes three DNA bases and the recognition features of ZFs can be manipulated. Since the FokI nuclease acts as a dimer, two ZFN molecules cooperate to cleave DNA. The pair of two ZFN-s recognize 18–24 base pairs. This allows for specific targeting of sequences in plant or mammalian genomes, including the human genome [3]. The double-strand break in DNA induces homology-directed repair in the presence of a suitable template [4]. This could offer a promising opportunity to cure monogenetic diseases [5]. However, due to a low level cytotoxicity ZFN-s were found to be not applicable in human therapy. Even though the FokI nuclease is a nonspecific nuclease, in the natural enzyme it is under negative allosteric control of the specific DNA-binding domain [6, 7]. In contrast, in the engineered ZFN-s this control is lost [8]. Thus, cytotoxicity might be caused by partial degradation of the protein in the cells. If the DNA-binding part is injured or lost, nonspecific cleavages of the chromosomal DNA are possible. The emerging new nucleases for genome editing are transcription activator-like effector nucleases (TALENs) and RNA-guided engineered nucleases [clustered regularly interspaced short palindromic repeat (CRISPR)–Cas (CRISPR-associated) system] [9, 10]. As an alternative, our goal is to design a safe, controlled ZFN with a positive allosteric control [11] to assure that the nuclease is only able to cleave DNA if the ZF-s are bound to the specific site. Based on its special structural characteristics in that its N- and C-termini cooperate in DNA-hydrolysis [12], we have selected the nuclease domain of colicin E7, a natural bacterial toxin (NColE7) for this purpose. This protein is expressed by *Escherichia coli* to protect the cell from related bacteria [13, 14].

In an attempt to design a controlled nuclease several issues have to be considered, such as (1) the determination of the essential functional parts of the enzyme (catalytic centre) that need to be kept intact, (2) identification of the parts of the protein that can be used to exert the control and (3) the question if the DNA binding part is necessary or if it can be replaced by ZF-s. The active centre of the NColE7 protein is an HNH motif at the C-terminus [15, 16]. One of the conserved histidines and two others coordinate to a divalent metal ion, which is a Zn^2+^-ion under physiological conditions [12, 17]. While binding to DNA is established with the major groove binding helices, the catalytic centre is located in the minor groove, to cleave the phosphodiester group yielding 5′-phosphate and 3′-OH ends [18]. The role of the metal ion is to coordinate to an oxygen of the scissile phosphate and to stabilize the negatively charged transition state. The nucleophile OH^−^ attacking the scissile phosphate was proposed to be generated from a water molecule coordinating to H545, which is the most conserved histidine in HNH proteins [19]. In order to maintain the nuclease activity of the newly designed enzyme, the HNH motif is clearly necessary. Crystallographic data as well as, biochemical and biophysical experiments in aqueous solution suggested that the metal binding site is preorganized in NColE7. We showed that deletions or modifications in the N-terminal sequence can destabilize the structure of NColE7 [12], and consequently, decrease metal- and DNA-binding affinity, thereby diminishing nuclease activity. This property can be utilized in the design of the positive control: the enzyme is active in the presence and has to be inactive in the absence of the N-terminal controlling unit. The role of the N-terminus of NColE7 has been studied in detail [20, 21]. The positively charged amino acids at the N-terminus are sterically close to the catalytic site. R447 is bridged with the Zn^2+^-ion by the scissile phosphodiester group. Thus it may take part in the binding, positioning and bending of the substrate DNA. Regardless of its exact function, the presence or absence of the N-terminus in the right position could be a way to control the nuclease. This makes NColE7 promising as a part of a controlled artificial nuclease.

Here, we explore by computational means the possibility to design a novel type of ZFNs by the fusion of NColE7 to a ZF protein (PDB code 1MEY) [22]. In a previous work we studied the specificity and thermodynamics of the ZF-binding to DNA [23]. In parallel, the study of the random mutations during the cloning process of cytotoxic NColE7 variants gathered information on the importance of various parts of the protein. Here, we started out from mutants that already had low activity to identify those mutation sites that result in nontoxic proteins. The mutations leading to nontoxic variants may indicate residues that contribute to the stabilization of the protein structure and assist the catalytic reaction. The hits revealed important interactions that should be maintained in the newly engineered nuclease.

## Methods

### Cloning and toxicity experiments

The pQE70 plasmid containing the genes of NColE7 and the Im7 immunity protein (a generous gift from prof. K.-F. Chak, Institute of Biochemistry and Molecular Biology, National Yang Ming University, Taipei, Taiwan) served as a template for the amplification of DNA segments including the gene of the native and mutated NColE7. The primers applied in PCR are collected in Table S1. The obtained fragments were cloned into a pGEX-6P-1 vector (GE Healthcare BioSci.) within the EcoRI and XhoI cloning sites. The plasmids were transformed into *E. coli* DH10B cells and spread on an LB/Amp (10 μg/ml ampicillin) plates. The colonies (usually 1–3 colony/100 μl transformed cell) were cultivated in LB/Amp (10 μg/ml ampicillin) solution, which was then sedimented and the plasmids were purified with the QIAGEN Mini Plasmid Purification kit. The purified plasmids containing the gene of the mutant proteins were used as templates in a further PCR with the pGEX sequencing primers, and the products were sequenced. Inactive mutants were selected by *E. coli* DH10B cells. Since NColE7 itself is toxic for the cells, in native bacteria it is coexpressed with its cognate immunity protein (Im7) [24, 25]. Here in the absence of Im7 gene the cloning procedure implied the selection of genes of nontoxic proteins.

### Construction of initial models

**Fig. 1:**
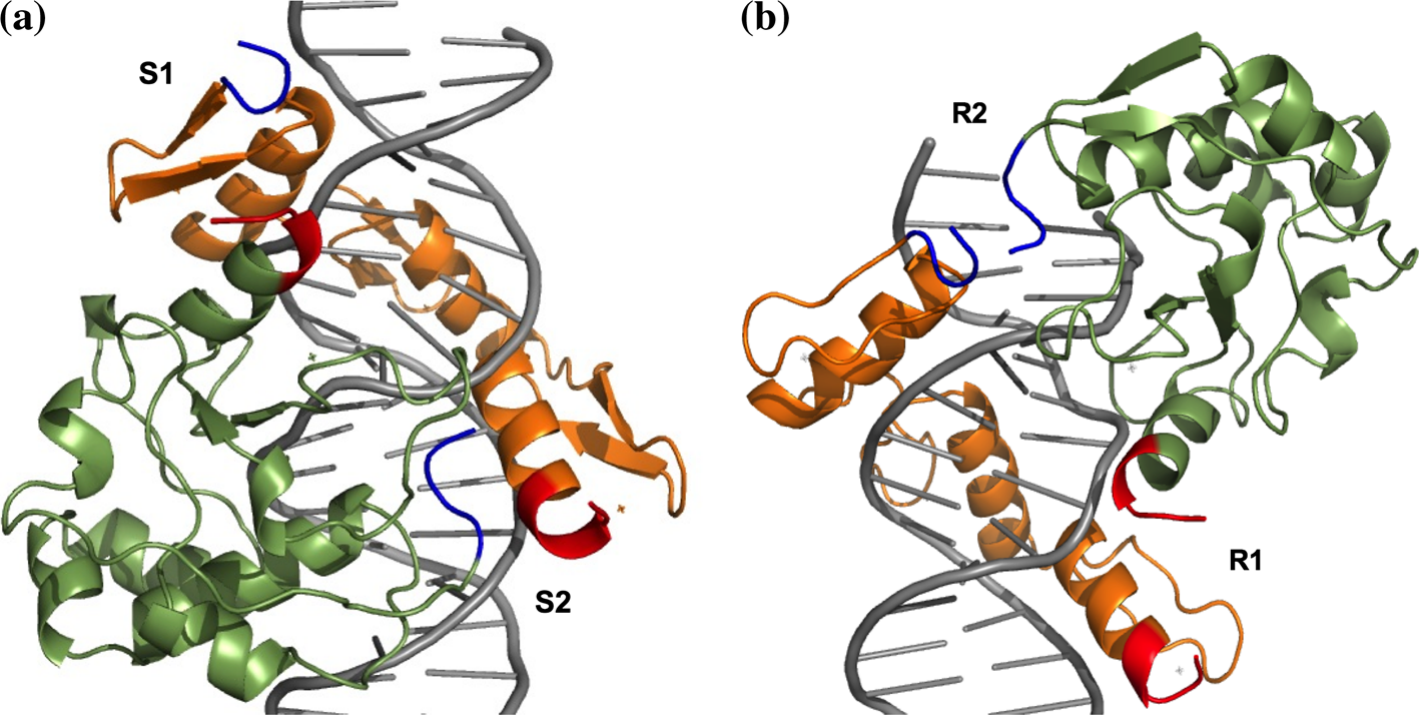
Two possible complexes for the design of a ZFN, in “straight” (**a**) and “reverse” (**b**) orientations. NColE7 in *green* and ZF protein consisting of three fingers in *orange*. The N- and C-termini of the proteins are marked in *blue* and *red*, respectively. The proposed links at positions S1, S2, R1 and R2 are indicated in Tables S2 and S3

The DNA/NColE7/ZF complexes were built by structural alignments based on the DNA chains of the DNA/NColE7 (2IVH, [26]) and DNA/ZF (1MEY, [27]) structures in PyMol. The initial models were constructed based on the following criteria: (1) The three ZF proteins bind DNA in the major groove; (2) NColE7 is also a major groove binding protein, however, its catalytic centre, the HNH-motif, acts in the minor groove; (3) The complex has to allow for the design of intramolecular allosteric regulation, which supposes that the protein takes one turn around DNA and the HNH motif is to be separated from the regulatory element by the ZF motifs. The proteins can fulfill these criteria in two possible orientations (Fig. 1). In the “straight” orientation the N-terminus of the ZF is close to the C-terminus of the nuclease thus, the linkers can be designed to bind the termini of the proteins to form a continuous circular sequence. In this case new termini can be chosen at any position of the NColE7 sequence. In contrast, in the “reverse” orientation the N-terminus of the ZF is close to the N-terminus of the nuclease, and the linkers have to connect the two proteins at other parts than their termini as two N-termini or two C-termini can not be fused. The fusion points of the sequences were selected based on sterical proximity of NColE7 and ZF parts in the initial reverse model.

In the alignment process all possible combinations of 5 or 8 base pair long parts of the sequence on both DNA chains were superimposed and the DNA originating from the DNA/NColE7 complex was removed. The structures were energy minimized using the GROMOS 45A4 force field [28] and the models with the lowest energy were selected. Out of the two DNA structures the specific site of the ZFs was kept in the final structure, and prolonged by repeating selected parts. Thus the initial models contained a DNA molecule with a sequence GAACTATGAGGCAGAACT (“straight” models) or AACTATGAGGCAGAACTATGAGG (“reverse” models) in complex with the ZF-s and NColE7 in close proximity, so that they could be joined with a suitable linker sequence.

### Linker design

The design of the linkers was carried out with the software package LoopX [29], an algorithm, designed to graft loop backbone coordinates into protein crystal. The underlying database of loop backbone coordinates (N, Cα, O, C) was constructed by in silico digestion of 14,525 protein crystal structures from the ASTRAL95 dataset (less than 95 % sequence identity). Linkers exhibiting appropriate length, end-to-end distance and RMSD of the respective anchor residues were identified from this database and included in the model as polyalanine chains. A position scan was carried out with FoldX [30], where all residues in a linker were mutated to the other 19 amino acids and changes in the free energy of folding were estimated. The sequences providing the energies of all designed loops are listed in Tables S2 and S3. Cysteins were excluded, because of their reactivity. Prolines were also excluded, as the computational methods seem to overestimate its stability due to covalent contributions; initially, one or more prolines were found in most of the linkers. Introducing extra prolines to the linker would introduce unwanted steric constraints to the structure. The structures were refined to avoid steric clashes, and the stability was estimated by FoldX.

**Fig. 2:**
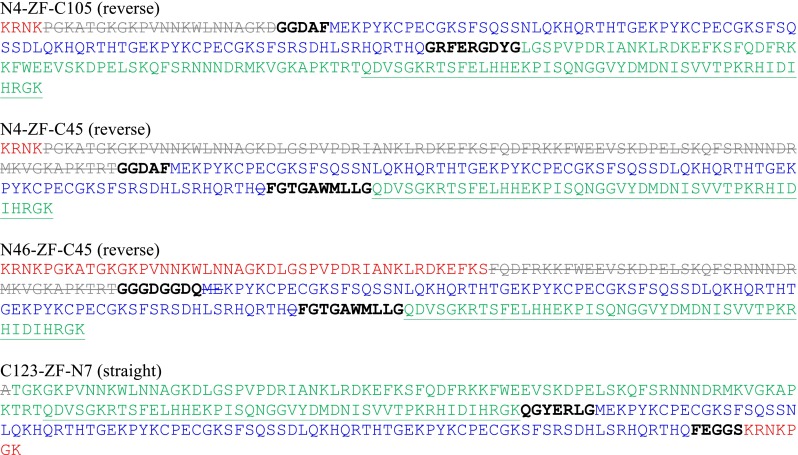
Sequences of the designed ZFNs. The ZF proteins are in *blue*, while NColE7 is divided into three parts: the original N-terminus (in *red*), the middle part not used in the model (in *grey*, *crossed out*) and the original C-terminal part (in *green*). The linkers are shown with *black bold letters*. The HNH motif of NColE7 is *underlined*. The N4–ZF–C105, N4–ZF–C45 and N46–ZF–C45 models are in the reverse orientation, while C123–ZF–N7 is the straight joint of protein sequences. For explanation of the names see the text

The four final models were constructed using all possible combinations of the best linkers, their sequences are shown in Fig. 2. The sequence of ZFs are unchanged in the models, except for cutting the last one or two residues when it was required for the linker design. NColE7 is divided into two parts: the N-terminus (N*X*) and the C-terminus (C*Y*), where *X* and *Y* refer to the number of residues involved in the model and *X* + *Y* is always less than the total number of amino acids in NColE7. The ZF sequence is inserted between these two parts. Accordingly, the “straight” models are named as C*Y*–ZF–N*X*, while the “reverse” models are named N*X*–ZF–C*Y*.

### MD simulations

MD simulations were carried out using the GROMOS11 suite of simulation programs [31], with the GROMOS force field, parameter set 45A4 [28]. The energy minimized starting structures of proteins, DNA molecules and complexes were centered in periodic rectangular boxes with a minimal solute-to-wall distance 0.9 nm. The box was filled with ~12,000 SPC water molecules with a minimal solute to solvent distance of 0.23 nm and subsequently minimized with the steepest descent algorithm, with a threshold of 0.1 kJ/mol to relax unfavorable interactions. Na^+^ or Cl^−^-ions were added to neutralize the system. Further water molecules at randomly selected positions were exchanged to Na^+^ and Cl^−^-ions to achieve a NaCl concentration of 0.2 M. The system was thermalized in 5 discrete simulation steps of 20 ps at increasing temperatures, followed by another 20 ps at 298 K. The MD simulations were run for 10 ns at 298 K (relaxation time 0.1 ps) using the weak-coupling method [32]. The pressure was maintained at 1 atm using the weak-coupling method and a relaxation time of 0.5 ps and an estimated isothermal compressibility of 4.575 × 10^−4^ (kJ/mol/nm^3^)^−1^. All bond lengths were constrained to their optimal value by the SHAKE algorithm [33] with a relative geometric accuracy of 10^−4^. During all calculations the Zn^2+^-ions were kept fixed using harmonic distance restraints: a Zn^2+^-N(His) interaction with an optimal bond length of 0.209 nm and a force constant of 14,710 kJ/mol/nm^2^, while the Zn^2+^-S(Cys) interactions with an optimal bond length of 0.231 nm and a force constant of 18,150 kJ/mol/nm^2^. The analysis of trajectories was done by various tools of the GROMOS++ suite [34].

## Results and discussion

### Investigation of colicin E7 mutations

We have previously shown that N-terminal point mutations decrease the nuclease activity of NColE7, but the mutants were still cytotoxic [21, 35]. The studied mutations included K446G, R447G, K449G, T454A, K458A and W464A in several combinations near the N-terminus of NColE7 (numbered 446–576). Cloning of the genes of these nucleases in pGEX-6P-1 vector without the gene of Im7 was not possible in the bacterial cells of *DH10B* *E. coli* strain, because of a leaking protein expression [36]. However, if the nuclease was inactivated by an additional random mutation, the cells could survive and copy the plasmid with the erroneous gene. Thus, the transformation process allowed for the selection of randomly mutated genes that encoded nontoxic proteins. Rare random mutations arose from the polymerase chain reaction used to construct the genes, using the DreamTaq (Fermentas) polymerase that has no proof reading activity. By cloning the WT enzyme we could identify severe mutations causing a reading frame shift. At the same time, it was possible to identify proteins with additional random point mutations by cloning genes of low activity N-terminal mutant proteins. The additional random mutations further decreased the low activity to a negligible level. The results are summarized in Tables 1 and S4.

**Table 1:** Mutations found in the cloning experiments of low activity NColE7 mutant genes

Multiplicity of mutations	Mutation
Single site	G473S, V476A, E488G, K525E, S535P, R538G, E542G, L543P, H545Y, D557E, D559 V, D559G, V564A, K567R
Double mutation	S474P + E508K F489L + S535P N560D + R568G
Triple mutation	G473C + E488D + L543P
Reading frame shift	IDIHRGK-LIFTEVNSS GK-VNSSSGRIVTD R574Stop

Single site mutations are point mutations, that occur along with the activity-decreasing designed N-terminal mutations, while double mutation sites occurred in pairs. Reading frame shifts are caused by the missing of one base. For more details see Table S4

**Fig. 3:**
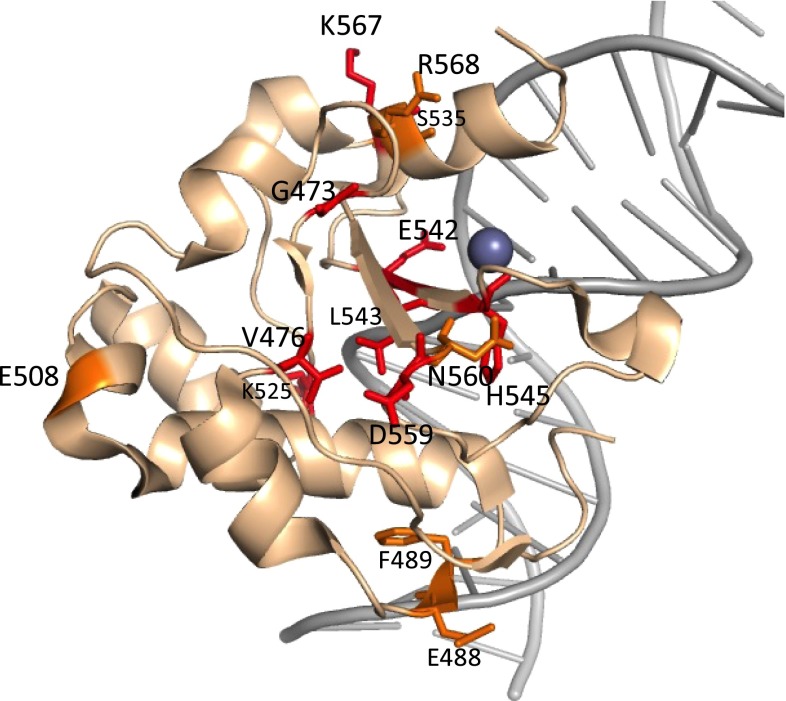
Location of the random mutations, as shown in the crystal structure 3FBD (D493Q–NColE7 in complex with a 18 bp DNA). The residues in red occur as single site mutations, while the *orange* ones are double site mutations

As shown in Fig. 3, several mutations occurred in the HNH-motif (S535, E542, L543, H545, N560, K567, R568), while there are other mutations further from the active site (G473, V476, E488, F489, E508, K525). The obtained random mutations indicate important interactions to consider in the process of the design of a new, NColE7-based ZFN.

### MD simulations: protein structure

**Fig. 4:**
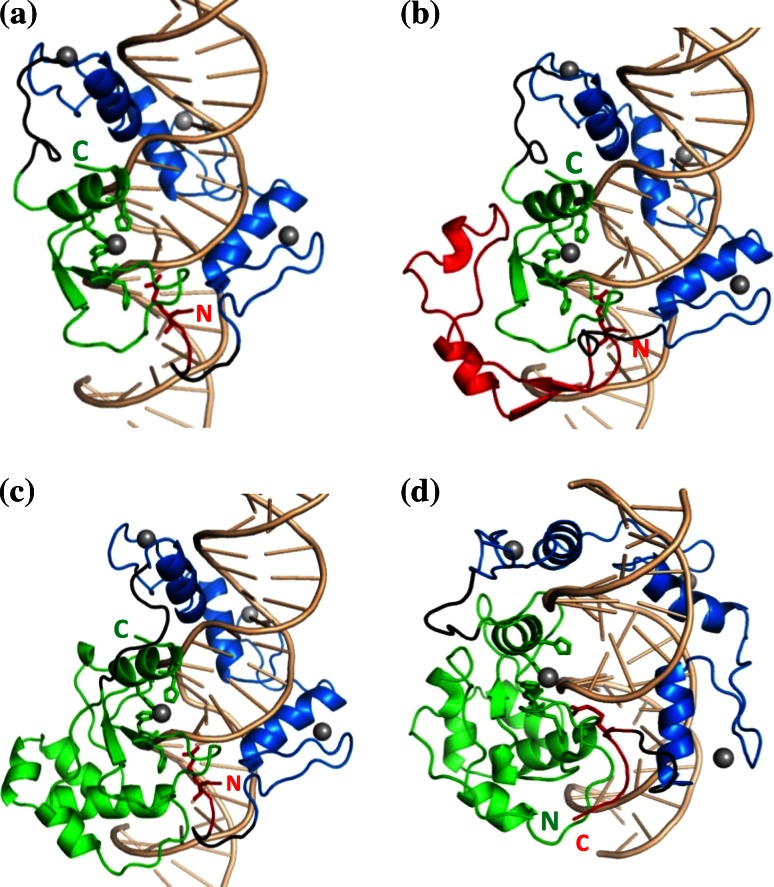
Designed ZFN structures, as starting points of MD simulations. The N-terminal part of NColE7 is shown in *red*, the C-terminal part in *green*, the ZF-s in *blue*, and the linkers are shown in *black*. The *grey* spheres indicate Zn^2+^-ions. **a** N4–ZF–C45 (reverse), **b** N46–ZF–C45 (reverse), **c** N4–ZF–C105 (reverse), **d** C123–ZF–N7 (straight). The C- and N-termini of the ZFNs are marked by “C” and “N”, respectively

In the linker design, as described in the “Methods” section, four models were selected for MD simulations. They are named as N*X*–ZF–C*Y* or C*Y*–ZF–N*X*, where *X* and *Y* indicate the number of N- and C-terminal NColE7 residues included in the model, respectively. The constructed models are shown in Fig. 4, while their sequences are shown in Fig. 2.

**Fig. 5:**
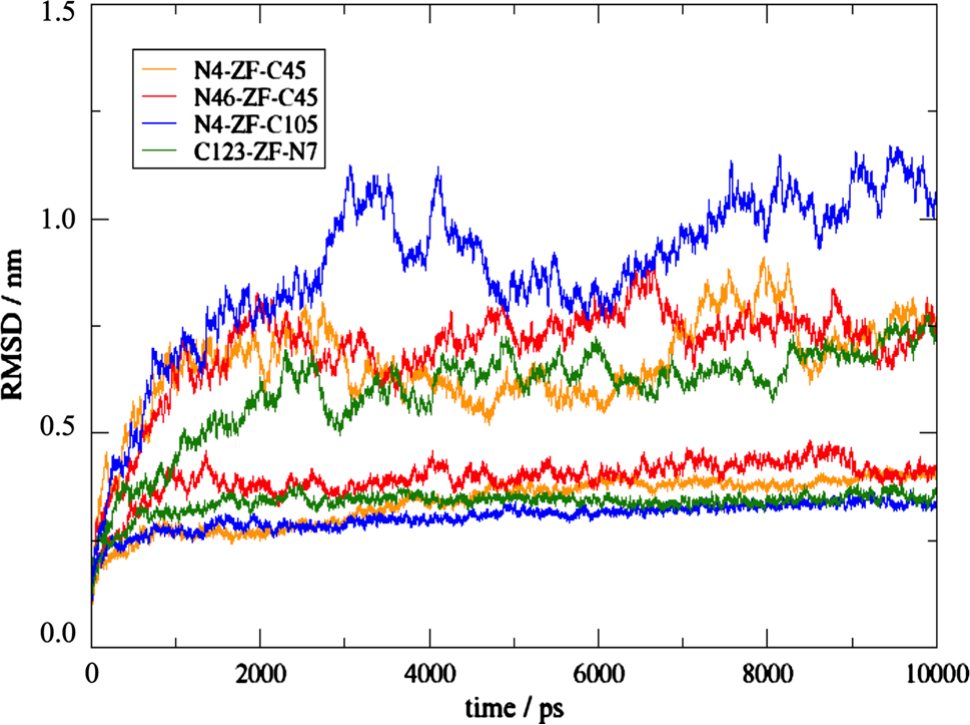
Atom-positional RMSD of the ZFN models in the simulations in complex with DNA (*lower curve* in each *color*) and in the control simulations of the proteins without the DNA (*higher curves* in each *color*)

10-ns MD simulations were performed to assess the stability and structural features of the designed proteins with and without DNA. The overall change of the structure compared to the starting structures is measured by the atom-positional root-mean-square deviation (RMSD) as a function of time (Fig. 5). The proteins in complex with DNA all converged towards values around 0.4 nm in the simulations, while in the control simulations without DNA they reached values up to 0.9 nm. The ZFN-s containing the major part of NColE7 as a continuous sequence, i.e. N4–ZF–C105 and C123–ZF–N7 had RMSD values that stayed closest to those in the reference simulation of NColE7, while N4–ZF–C45 and N46–ZF–C45 showed increased RMSD values. Obviously, the initial structures used as references will resemble the continuous NColE7 structure most and a slightly larger structural rearrangement as observed for models N4–ZF–C45 and N46–ZF–C45 does not disqualify these designs a priori. None of the free ZFN proteins reached equilibrium within 10 ns, because the cyclic shaped molecules continued to change their tertiary structure. This could be detected also by the changes in the radius of gyration (Fig. S1) attributed to the flexible linkers and not to the protein domains themselves. The ZFs behave similarly in all four models: they had a stable structure as seen in snapshots and low atom-positional root-mean-square fluctuations (RMSF <0.2 nm). In contrast, the nuclease part without the DNA substrate was less stable. The DNA structure was stabilized in a similar manner by all four ZFN proteins (Fig. S2).

The protein-DNA interaction energies were compared in Table 2. The convergence of the interaction energies is shown in Fig. S3. The N4–ZF–C105 model shows the strongest interaction with DNA. This result is expected among the reverse models, since this model contains the most intact NColE7 domain. Even the straight model has weaker interaction with the substrate, which can be explained by the more flexible loop in N4–ZF–C105 and the C-terminal position of HNH motif, similarly to the case of natural NColE7.

The simulations were also compared to the experimentally determined residues that were deemed to be important for the nuclease activity. In each case, particularly in the “reverse” approach of model building, certain parts of the nuclease were necessarily removed in the linking procedure. Table 3 shows how many of these residues were determined to be important according to the toxicity experiments. The MD simulations confirmed the expectations based on the statistics of missing residues, in that models N4–ZF–C105 and C123–ZF–N7 behave most similarly to NColE7.

**Table 2:** Interaction energies calculated as the sum of van der Waals and electrostatic interaction energies between the protein and DNA molecules

Model	Average interaction energy (kJ/mol)
N46–ZF–C45	−6,234.9 ± 38.9
N4–ZF–C45	−6,108.5 ± 33.2
N4–ZF–C105	−7,368.8 ± 68.3
C123–ZF–N7	−6,383.7 ± 64.4

**Table 3:** Comparison of the ZFN sequences with the results of the low activity NColE7 mutagenesis: the listed residues are considered as important due to mutations in the experimental assay, but are missing from the sequence of the indicated models

Model	Missing from NColE7	Missing single mutation sites	Missing double mutation sites	Missing triple mutation sites
N4–ZF–C45	450–531 (82 res)	G473 V476 E488 K525 S535	S474 F489 E508	G473 E488
N46–ZF–C45	492–531 (40 res)	K525	E508	–
N4–ZF–C105	450–472 (23 res)	G473	–	–
C123–ZF–N7	453 (1 res)	–	–	–

### MD simulations: catalytic centre and possibility of the allosteric control

**Fig. 6:**
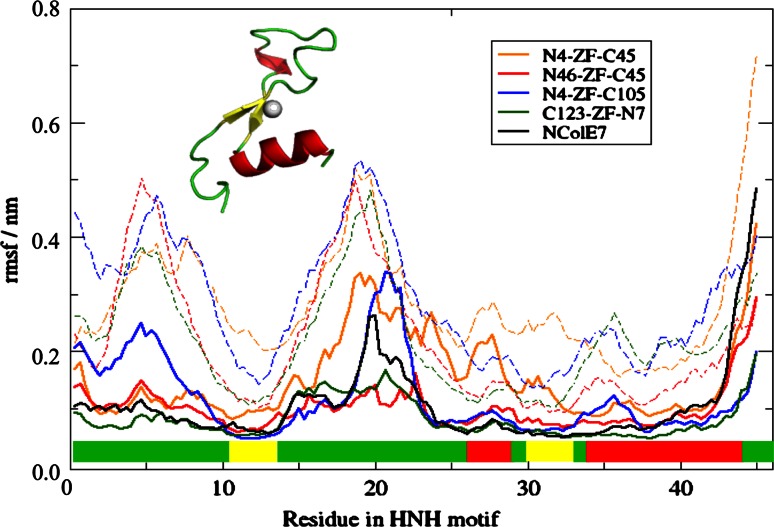
Atom-positional RMSFs of residues in the HNH motif of ZFN-s and NColE7 as a reference. The residue numbers are shifted to 1–45 in all models for comparison, corresponding to the HNH residues 532–576 in the NColE7 numbering. *Dashed lines* indicate the control simulations for proteins without DNA. The secondary structural elements of HNH motif are marked above the *x axis*, showing the loops in *green*, α-helices in *red* and β-sheets in *yellow*

For the designed ZFN proteins not only the overall structural stability, but also the proper function of the catalytic centre, i.e. the HNH motif is essential. The secondary structure of the HNH motif was stable in the simulations of ZFN-s with DNA substrate, as analyzed by DSSP [37] (Figs. S4–S6). The atom-positional RMSFs of the HNH residues are compared in Fig. 6. N4–ZF–C45 had the least stable HNH-motif both in the DNA-bound and free form. This is in agreement with our previous study on the HNH motif itself [38], where we showed that its isolated structure is not stable and its zinc-binding is weak. The HNH-motif of C123–ZF–N7 has the most similar features to that of NColE7—as expected, since this protein has the most complete nuclease domain. Despite the small deviations, all four models retained the β-sheets and the α-helix in the HNH-motif.

**Fig. 7:**
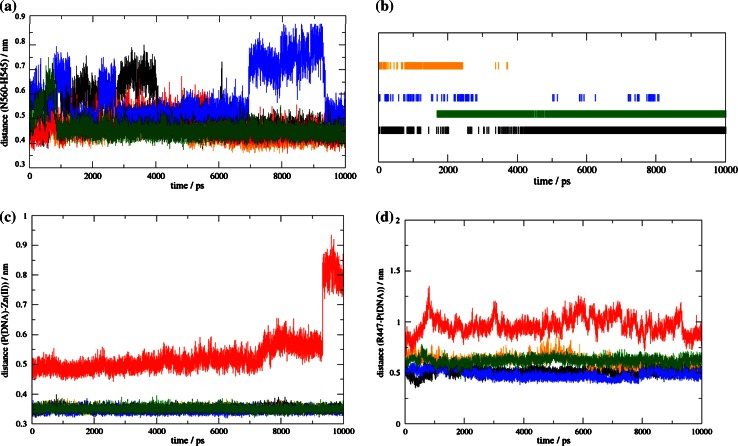
**a** Distance of residues corresponding to N560 (CG) and H545 (CA) in NColE7 as a function of time. **b** Presence of H-bond between the residues corresponding to H545 and V555 in NColE7. **c** Catalytic distances as a function of time: Zn^2+^-O (at the scissile phosphate) and **d** R447(NE)-P (scissile phosphate). In all panels, NColE7 is represented by *black curves*, N4–ZF–C45 by *orange*, N46–ZF–C45 by *red*, N4–ZF–C105 by *blue* and C123–ZF–N7 by *green*

For nuclease activity, also the hydrogen-bond structure in the loop between the antiparallel β-sheets is required. The interactions within the HNH motif, that are deemed to be important for catalysis, were also analyzed. N560 (NColE7 numbering) is a conserved residue in the HNH proteins, and it interacts with H545, the general base activating a water molecule to form the nucleophilic OH^−^-ion. The equilibrated distances between these two residues (measured between the CG atom of N560 and CA atom of H545) were ~0.45 nm in all models, except for N4–ZF–C105 (Fig. 7a), where it increased in the 7,000–9,000 ps interval due to a conformational change of N560. The H545–V555 H-bond maintained the orientation of the H545 general base. This H-bond was stable only in NColE7 and the C123–ZF–N7 model (Fig. 7b). During the catalytic reaction the Zn^2+^-ion of the nuclease domain binds the oxygen of the DNA scissile phosphate group. The Zn^2+^-O distance was found to be stable as a function of time for each of the models except for N46–ZF–C45, where the coordination bond was lost (Fig. 7c).

The control mechanism of the designed ZFN would be assured by the positively charged sequence, originally found at the N-terminus of NColE7 (“KRNK”). Particularly, R447 is important, as it stretches out its side-chain towards the scissile phosphodiester group. The analysis of the distances between these two residues as a function of time showed that N46–ZF–C45 is less probable to exert nuclease activity (Fig. 7d). It is important to note that all the other models showed an Arg-P distance suitable for an allosteric control mechanism. Concerning this distance, the N4–ZF–C105 model is the most optimal, confirming that it is preferable to keep the controlling loop at the N-terminus and the catalytic centre at the C-terminus.

**Fig. 8:**
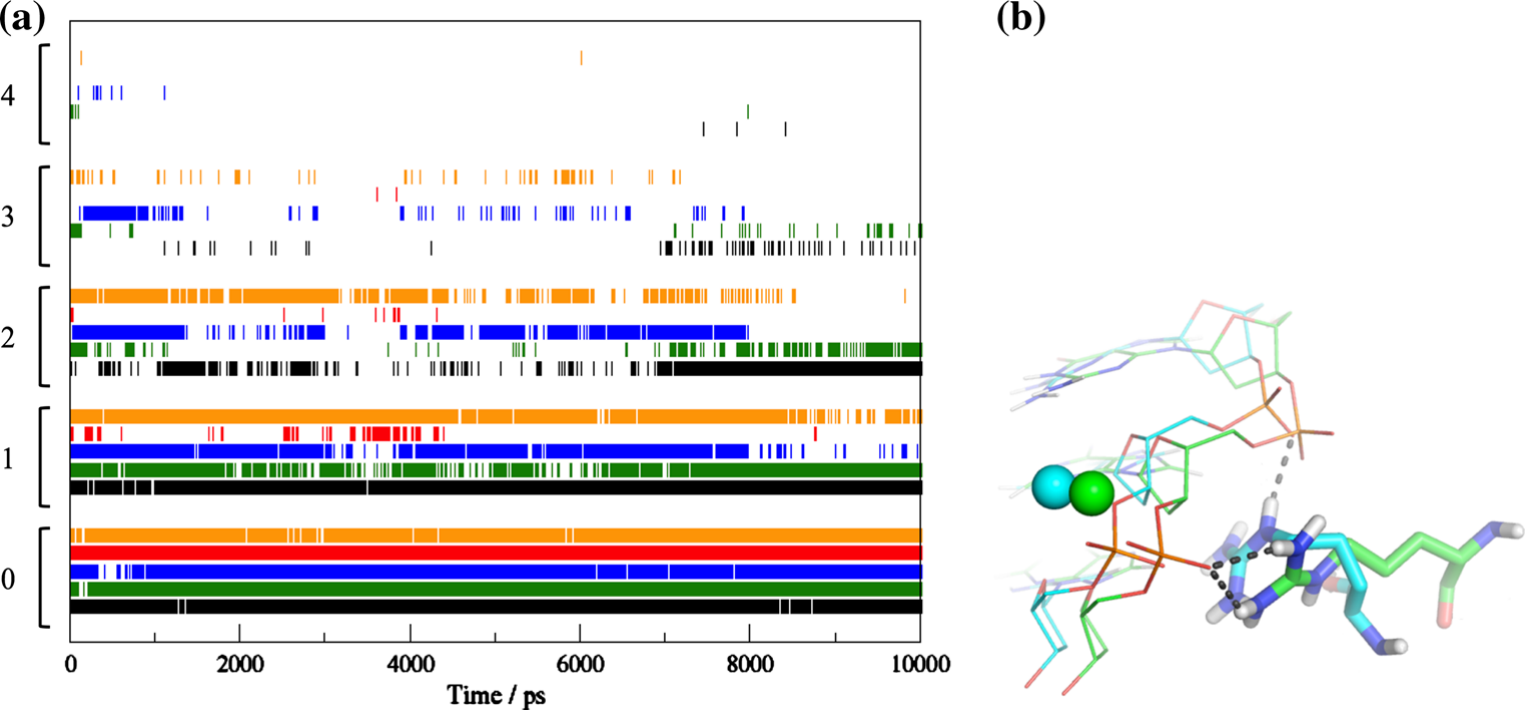
**a** The number of H-bonds between the arginine corresponding to R447 in NColE7 and the DNA atoms in the different models as a function of time. Numbers on the *y axis* indicate how many hydrogen bonds were found at a given time. NColE7 is in *black*, N4–ZF–C45 in *orange*, N46–ZF–C45 in *red*, N4–ZF–C105 in *blue* and C123–ZF–N7 in *green*. **b** The interactions of the controlling Arg at 2,000 ps (in* green*) and 8,500 ps (in *blue*) in the N4–ZF–C45 simulation

The crucial role of the N-terminus of NColE7 in the catalytic reaction has been studied in detail. Possible roles that were proposed include the binding and electrostatic activation of the DNA substrate and/or preventing the reverse reaction [20, 39]. The interactions between R447 and DNA reflect the ability of this residue to assist the catalysis in the ZFN models. Figure 8a shows the number of H-bonds between the atoms of the residue corresponding to R447 in NColE7 and DNA atoms. C123–ZF–N7 has the most similar dynamics of R447-DNA interactions to NColE7, showing three hydrogen bonds with a reasonable probability at the end (7–10 ns) of the simulation. The Arg in N4–ZF–C105 and N4–ZF–C45 also shows an extensive H-bond network. Notably, the Arg in N4–ZF–C45 binds to the same phosphate group as NColE7 in the beginning, but changes to the neighboring phosphate after 6,500 ps (Fig. 8b) indicating that the truncation of the nuclease structure has high impact on the function. N46–ZF–C45 forms Arg-DNA H-bonds only a few times during the simulation, which is the result of more severe structural changes after dividing the nuclease over two domains of the constructed ZFN.

## Conclusions

In the process of the design of NColE7-based ZFNs a novel type of control was introduced. Namely, NColE7 was divided into two parts using its special features: the HNH-motif at the C-terminus of the protein is only functional in the presence of the positively charged N-terminal sequence. These parts are linked to different ends of the ZF array. Therefore, the newly modeled nucleases should be able to exhibit their hydrolytic activity only upon ZF binding to the specific DNA recognition sequence and as a result, the controlling loop and the HNH motif become in suitable proximity. By establishing such a control, we avoid the risk that partially degraded proteins maintain nonspecific activity, leading to cytotoxicity.

Four ZFN models of this kind have been computationally designed and subjected to molecular dynamics simulations. Three models (N4–ZF–C45, N4–ZF–C105 and N46–ZF–C45) had, similarly to NColE7, the allosteric sequence at the N-termini, the HNH-motif based parts at the C-termini and the ZF array in between. A fourth model (C123–ZF–N7) rather placed the allosteric sequence at the C-terminus and the HNH-motif at the N-terminus. This latter model simply connected the termini of the NColE7 to the ZFs. This approach allows for easy redesign, however, its drawback is, that the termini are exchanged. As a result of this the distance between the Arg (corresponding to R447 in NColE7) and DNA increased and the number of H-bonds involving Arg decreased during the simulation. Also, the structure of the isolated HNH motif was less stable in comparison to the oppositely built model, i.e. N4–ZF–C105 as predicted by DSSP. Consequently, the ZFN molecules keeping the original termini seem to be more promising. The most complete model among these three is the N4–ZF–C105, which exhibits almost all the interactions predicted to be important in the mutation experiments. All features of this model were similar to NColE7 with a small difference in the loop between the β-strands of its HNH-motif, which was more flexible based on the RMSF profile and the characteristic H545–N560 distance. These changes probably arose from the interaction of the loop both with the bulky part of NColE7 and with the newly designed linker. The change of the loop structure could hamper the nuclease activity, since this part plays an important role in the reaction, promoting the generation of the nucleophilic OH^−^. The nuclease in the N4–ZF–C45 model is truncated and contains only the N-terminal KRNK sequence and the HNH-motif. It was previously shown experimentally, that the HNH motif itself does not fold correctly, and the lower stability can also be seen in the control simulations for proteins without DNA. However, this model may still be functional as the interaction with DNA could induce the correct structure, since it behaves well in the simulations with DNA. The N46–ZF–C45 model is tempting in terms of strict conformational regulation of the ZFN, as the two independent parts of NColE7 have to fold together while the ZF-s bind around the specific DNA. However, even though the protein is stable, the catalytic properties of this model in the simulations were significantly worse than in the other models.

In summary, the three models including C123–ZF–N7, N4–ZF–C105, N4–ZF–C45 are promising in terms of catalytic activity, and the first two of these also in their structural robustness. The nucleases designed here may provide an alternative to the FokI-based artificial nucleases, and will be experimentally studied in the future.

## Electronic supplementary material

Below is the link to the electronic supplementary material.

Supplementary material 1 (PDF 852 kb)
