# Supplementary material for: Design of a colicin E7 based chimeric zinc-finger nuclease
Source: J Comput Aided Mol Des. 2014 Jun 22;28(8):841–50. doi: 10.1007/s10822-014-9765-8 (PMC4104000; doi:10.1007/s10822-014-9765-8)
Supplement: Supplementary file 1 — Supplementary material 1 (PDF 852 kb) [file 10822_2014_9765_MOESM1_ESM.pdf]

Electronic Supplementary Information for the manuscript:

Design of a colicin E7 based chimeric zinc-finger nuclease

Eszter Németh[a], Gabriella K. Schilli[a], Gábor Nagy[b], Christoph Hasenhindl[c], Béla Gyurcsik*[a],[d], Chris Oostenbrink*,[b],

[a]Department of Inorganic and Analytical Chemistry, University of Szeged, Dóm tér 7, H-6720 Szeged, Hungary

[b]Institute of Molecular Modeling and Simulation, BOKU - University of Natural Resources and Life Sciences, Muthgasse 18, A-1190 Vienna, Austria

[c]Christian Doppler Laboratory for Antibody Engineering, Department of Chemistry, Vienna Institute of BioTechnology, BOKU - University of Natural Resources and Life Sciences, Muthgasse 18, A-1190 Vienna, Austria

[d]MTA-SzTE Bioinorganic Chemistry Research Group of Hungarian Academy of Sciences, Dóm tér 7, H-6720 Szeged, Hungary

Table S1. Sequence of primers used for the mutagenesis.

primer	Sequence
GST/2	5'-GGTGATCATGTAACCCATCCTGACTTCATG-3'
NColE7-5'	GGAATTCAAACGGAATAAGCCAGGGAAG
KGNK-5'	GGAATTCAAAGGGAATAAGCCAGGGAA
KGNG-5'	GGAATTCAAAGGGAATGGGCCAGGGAAGGCAACAGG
GGNK-5'	GGAATTCGGAGGGAATAAGCCAGGGAAGGCA
GGNG-5'	GGAATTCGGAGGGAATGGGCCAGGGAAGGCAACAGG
W/A-3'-2.	5'-GCATTATTTAAGGCCTTATTATTGACAGGTTTTCCTTTACC-3'
T/A-5'-2.	5'-GGAAGGCAGCTGGTAAAGGAAAACCTGTCAATAATAAG-3'
T/A-5'-3.	5'-GGAAGGCAGCTGGTAAAGGAGCCCCTGTCAATAATAAG-3'
T/A-3'-2.	5'-CTTTACCAGCTGCCTTCCCTGGCTTATTCCGTTT-3'

Table S2. Linker sequences and energies of the structures with all modeled loops in the straight model. Every linker in the table corresponds to an individual hit in the linker search, and the corresponding best sequence is given. The black bold linkers are selected for the models.

straight							
S1		S1		S2		S2	
linker	energy kcal/mol	linker	energy kcal/mol	linker	energy kcal/mol	linker	energy
FYYDDI	114,87	**QGYERLG**	**113,20**	**FEGGS**	**123,78**	EGGKGG	125,88
LGQMQG	115,25	LGRGYKG	119,12	DGGDS	125,24	FNGKDG	127,24
FEFDDE	115,98	DGLGQKM	119,22	AGGNG	126,60	GEHTES	127,37
DYEMKI	117,50	EEFLAGL	119,26	GEGGV	127,01	LERMNT	127,59
AFEFGG	118,08	FEFFKWL	119,82	GLEFG	127,05	LEDEEI	128,42
MGMKFM	118,57	AWMDGRY	121,85	GGEGV	127,78	SRFGFN	131,24
FGLEKM	118,88	GDEEDEG	125,61	DEEEI	128,34	SGFAFY	134,34
FGMYEM	119,19	MFGEEWS	129,43	DGGGL	128,37	AEEEET	135,03
GGDEID	119,19	GGFKDGL	131,21	FEGGS	128,72	GQRFMN	135,70
LGKFKG	119,95	MGQYQRK	132,68	GEGET	128,94	GGGEGS	140,71
MEETEI	120,33			AGGYG	129,25	GGGDSN	141,82
FGDREI	120,46			DEEEI	130,09	GFGGEY	142,33
MGRYFA	121,36			GGEGG	131,44	GGGFFG	151,00
KGREEM	122,30			GGGKV	132,35		
MGREEM	122,66			GEGGS	132,60		
MGDEYD	123,38			GEFKA	133,89		
WGIKEG	124,39			GEADA	135,65		
GRGFMS	125,07			GGGYG	136,76		
WGRSKM	125,07			GLQGG	137,26		
MQDEED	125,38			EGFKL	137,69		
GDGDKW	131,23			EGGGA	138,38		
				GDADT	139,06		
				DGLKY	141,44		
				QGGGV	142,59		
				DFKGG	142,60		
				GGGGG	160,16		
				SGGGG	160,28		
				GGGDG	160,74		
				GGGEG	168,64		
				GGGGN	169,64		

Table S3. Linker sequences and energies of the structures with all modeled loops in the reverse model. Every linker in the table corresponds to an individual hit in the linker search, and the corresponding best sequence is given. The black bold linkers are selected for the models.

reverse							
R1/1		R1/2		R2/1		R2/2	
linker	energy kcal/mol	linker	energy kcal/mol	linker	energy kcal/mol	linker	energy
GGGGGGGS	165,74	**GGDAF**	**73,35**	**FGTGAWMLLG**	**63,79**	**GRFERGDYG**	**78,06**
**GGGDGGDQ**	**166,21**	GFWEQ	74,23			QQFEEQQKG	78,36
GGGGGGGD	184,53	DGDEM	74,83			GGFQREERG	82,16
GGGGGGGE	198,01	GGDGG	76,19			GNFEKEYRG	82,73
GGAGGGGG	200,92	GDDGE	76,81			QIDLFHQFG	87,62
GGGGGGGD	202,27	GDDEE	77,03				
GGGGGGGG	219,53	GDDEG	77,60				
GGGGGGGG	252,27	GYDEF	78,00				
		GGGDF	80,15				
		GVDEF	80,19				
		GGDGE	83,12				
		GGFEQ	83,24				
		GGWEE	83,37				
		GGGGE	84,64				
		GDFEG	85,46				
		GGIDE	94,33				

Table S4. Detailed result of the mutagenesis. The first column contains the designed N-terminal mutations that diminish the nuclease activity and the second column shows the random mutations that completely cancel the low activity.

designed mutations	random mutations
R447G/K449G	H545Y
	G473S
	G473C/E488D/L543P
	L543P
	E542G
K446G/R447G	S535P
	E488G-R574Stop
	V564A
	E542G
	D559G
K446G/R447G/K449G	IDIHRGK-LIFTEVNSS
	GK-VNSSSGRIVTD
	D557E
	K567R
	S474P/E508K
	V476A
	K525E
T454A	N560D/R568G
	F489L/S535P
T454A/K458A	R538G
K458A/W464A	D559V

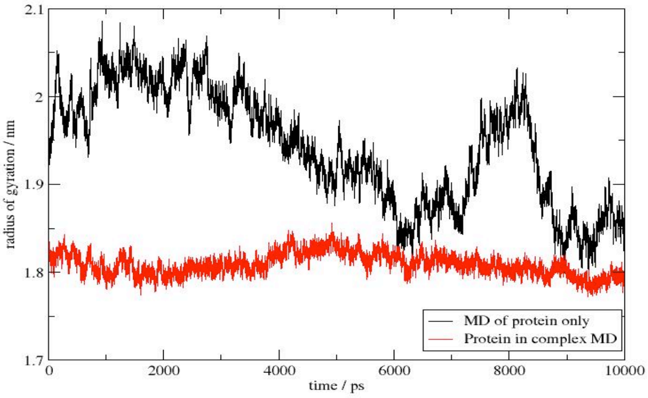

Fig. S1. Radius of gyration in the simulation of N4-ZF-C45 in complex with and in absence of DNA.

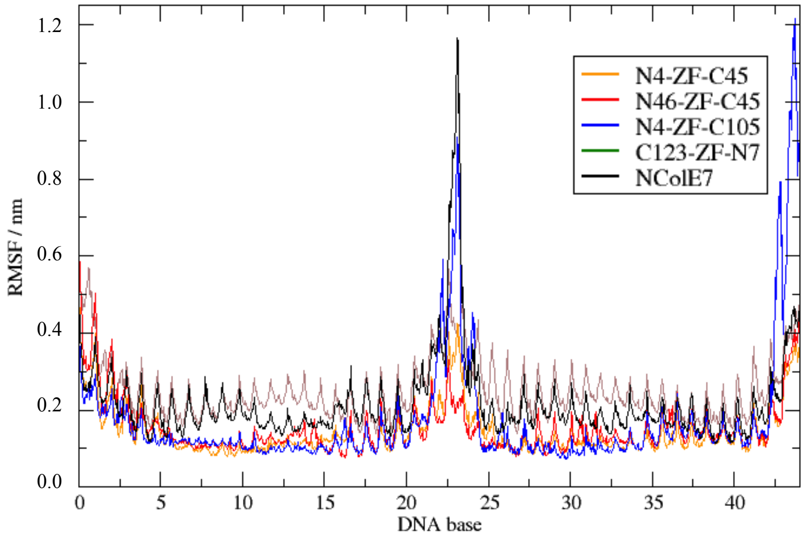

Fig. S2. Atom-positional root-mean-square fluctuations (RMSF) of DNA bases in both strands, in the reverse models. DNA bases 1-23 are paired to bases 46 – 24 in the structures.

B)

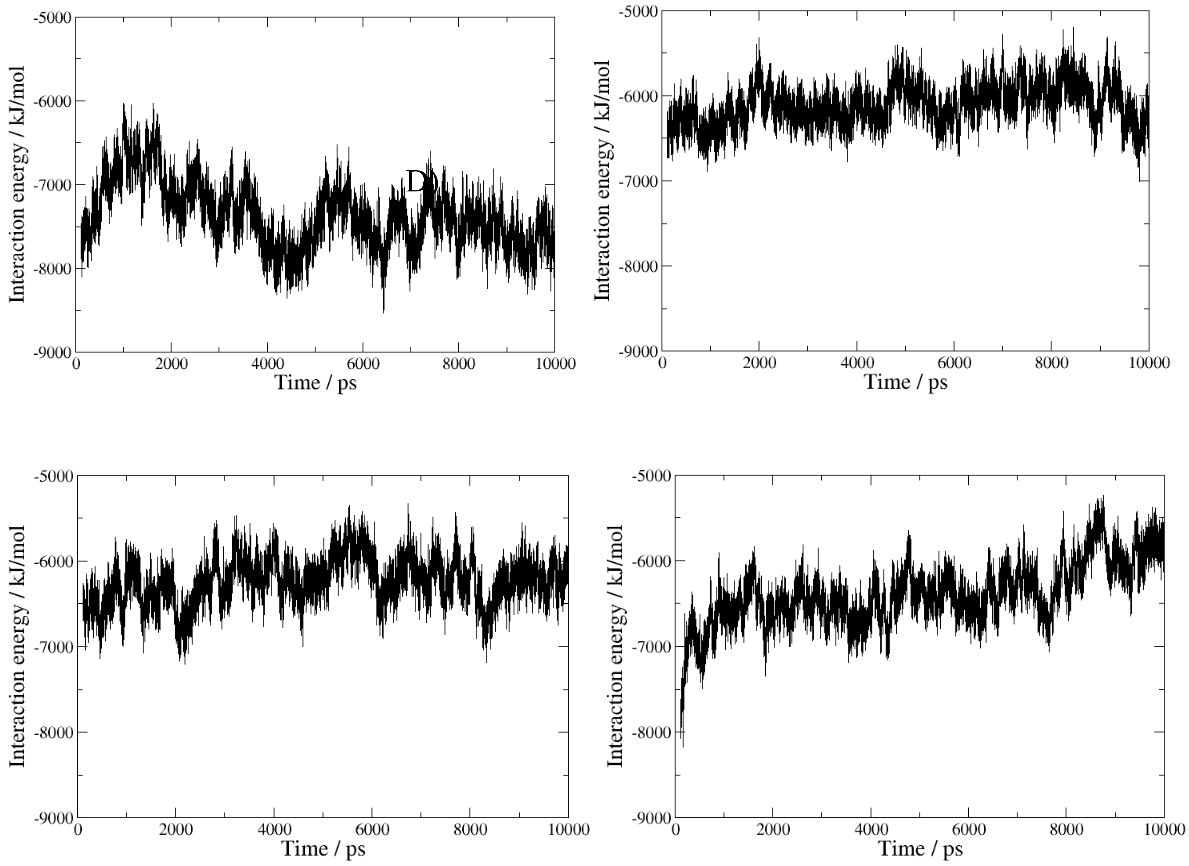

Fig. S3. Interaction energy between the proteins and DNA a) N4-ZF-C105 b) N4-ZF-C45 c) N46-ZF-C45 d) C123-ZF-N7

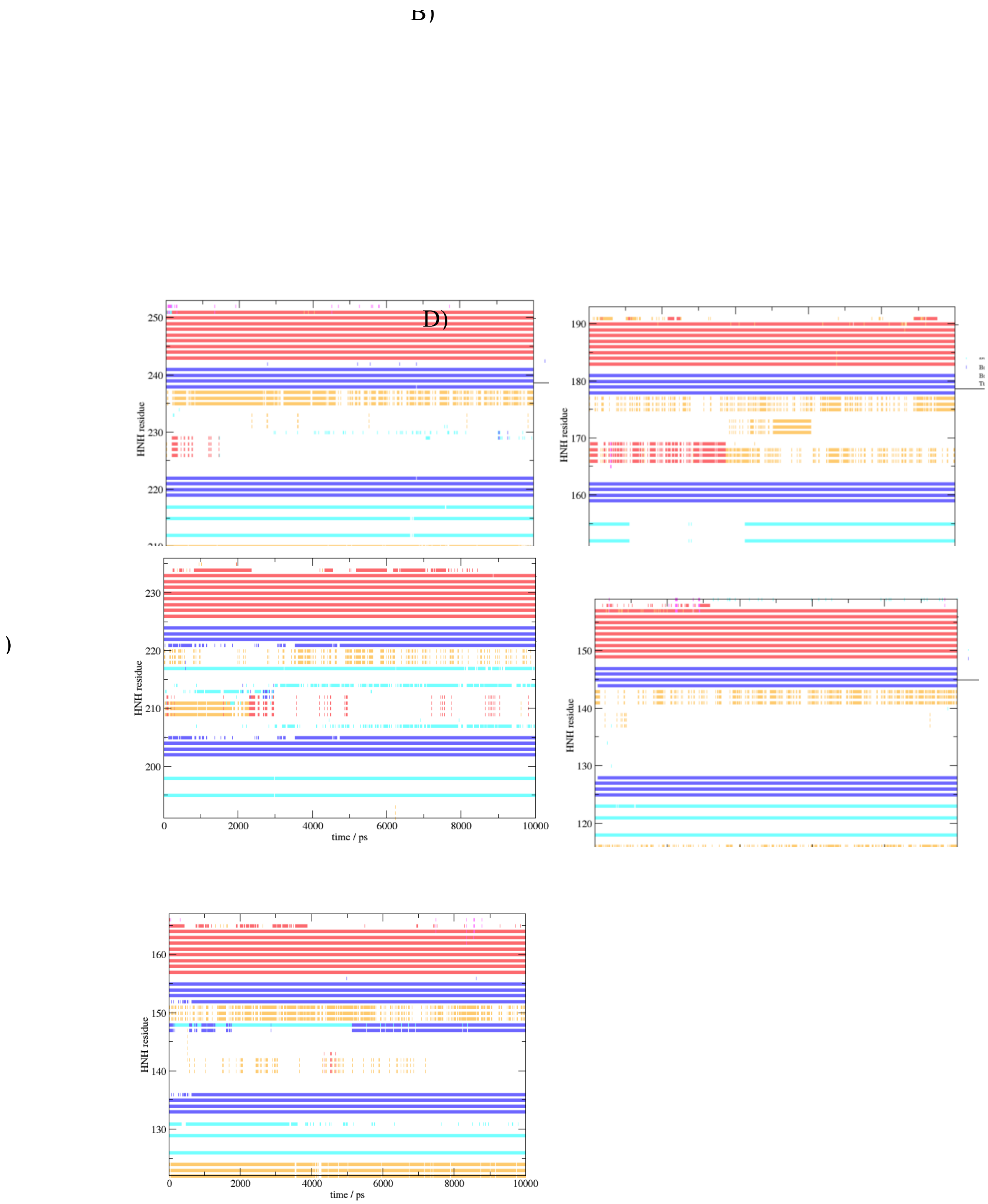

Fig. S4. Secondary structure elements of the HNH motives in NColE7 and the ZFN models, in the simulations with DNA: a) N4-ZF-C105 b) N4-ZF-C45 c) N46-ZF-C45 d) C123-ZF-N7 e) NColE7. The 3-type helix is in yellow, the 4-type helix in red, β-strands are in blue and β-bridges in light blue.

)

B)

D)

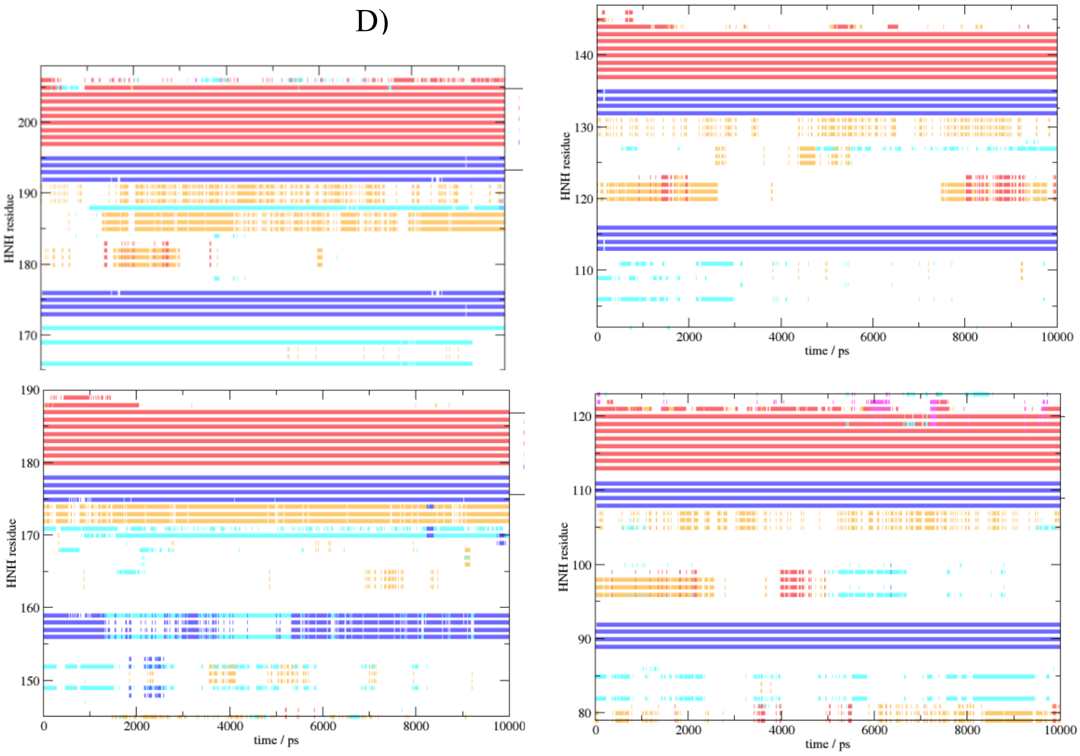

Fig. S5. Secondary structure elements of the HNH motives in NColE7 and the ZFN models, in the simulations of free proteins without DNA: a) N4-ZF-C105 b) N4-ZF-C45 c) N46-ZF-C45 d) C123-ZF-N7. The 3-type helix is in yellow, the 4-type helix in red, β-strands are in blue and β-bridges in light blue.

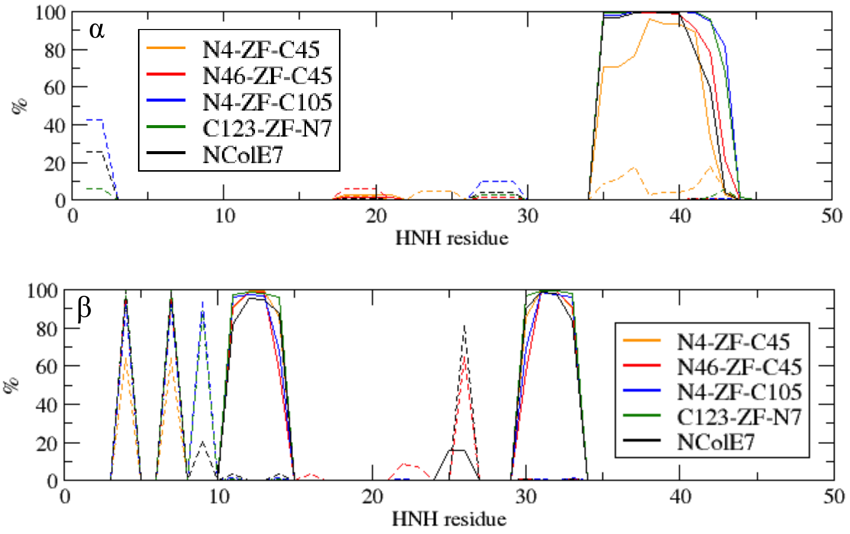

Figure S6. Presence of secondary structure elements as a percent of time for each HNH residues in the different models.
